# Could Changes in the Agricultural Landscape of Northeastern China Have Influenced the Long-Distance Transmission of Highly Pathogenic Avian Influenza H5Nx Viruses?

**DOI:** 10.3389/fvets.2017.00225

**Published:** 2017-12-19

**Authors:** Marius Gilbert, Diann J. Prosser, Geli Zhang, Jean Artois, Madhur S. Dhingra, Michael Tildesley, Scott H. Newman, Fusheng Guo, Peter Black, Filip Claes, Wantanee Kalpradvidh, YeunKyung Shin, Wooseog Jeong, John Y. Takekawa, Hansoo Lee, Xiangming Xiao

**Affiliations:** ^1^Spatial Epidemiology Lab (SpELL), Université Libre de Bruxelles, Brussels, Belgium; ^2^Fonds National de la Recherche Scientifique (FNRS), Brussels, Belgium; ^3^Patuxent Wildlife Research Center, United States Geological Survey, Beltsville, MD, United States; ^4^Department of Microbiology and Plant Biology, Center for Spatial Analysis, University of Oklahoma, Norman, OK, United States; ^5^School of Life Sciences, University of Warwick, Warwick, United Kingdom; ^6^Food and Agriculture Organization of the United Nations, Addis Ababa, Ethiopia; ^7^Regional Office for Asia and the Pacific, Food and Agriculture Organization of the United Nations, Bangkok, Thailand; ^8^Animal and Plant Quarantine Agency, Ministry of Agriculture, Food and Rural Affairs, Gimcheon, South Korea; ^9^San Francisco Bay Estuary Field Station, Western Ecological Research Center, United States Geological Survey, Vallejo, CA, United States; ^10^Richardson Bay Audubon Center & Sanctuary, Tiburon, CA, United States; ^11^Korea Institute of Environmental Ecology, Daejeon, South Korea; ^12^Institute of Biodiversity Science, Fudan University, Shanghai, China

**Keywords:** avian influenza, land use change, disease ecology, agriculture and public health, spatial epidemiology

## Abstract

In the last few years, several reassortant subtypes of highly pathogenic avian influenza viruses (HPAI H5Nx) have emerged in East Asia. These new viruses, mostly of subtype H5N1, H5N2, H5N6, and H5N8 belonging to clade 2.3.4.4, have been found in several Asian countries and have caused outbreaks in poultry in China, South Korea, and Vietnam. HPAI H5Nx also have spread over considerable distances with the introduction of viruses belonging to the same 2.3.4.4 clade in the U.S. (2014–2015) and in Europe (2014–2015 and 2016–2017). In this paper, we examine the emergence and spread of these new viruses in Asia in relation to published datasets on HPAI H5Nx distribution, movement of migratory waterfowl, avian influenza risk models, and land-use change analyses. More specifically, we show that between 2000 and 2015, vast areas of northeast China have been newly planted with rice paddy fields (3.21 million ha in Heilongjiang, Jilin, and Liaoning) in areas connected to other parts of Asia through migratory pathways of wild waterfowl. We hypothesize that recent land use changes in northeast China have affected the spatial distribution of wild waterfowl, their stopover areas, and the wild-domestic interface, thereby altering transmission dynamics of avian influenza viruses across flyways. Detailed studies of the habitat use by wild migratory birds, of the extent of the wild–domestic interface, and of the circulation of avian influenza viruses in those new planted areas may help to shed more light on this hypothesis, and on the possible impact of those changes on the long-distance patterns of avian influenza transmission.

The highly pathogenic avian influenza (HPAI) H5N1 virus initially emerged in China in 1996, but it was only in 2003–2004 that it started to spread trans-nationally in Southeast Asia (1). In 2005–2006, it spread across the Eurasian continent into Europe and south to sub-Saharan Africa. The virus did not persist in most countries where it had been introduced, but it did so in a few countries such as China, Vietnam, Indonesia, and Egypt where high densities of chickens, ducks, and live-poultry markets created conditions favoring long-term persistence (2). Since its first detection, the virus has evolved, and these changes have been traced in the H5 hemagglutinin gene through a nomenclature describing clades and sub-clades (3).

From 2009 to 2014, H5N1 apparently reassorted with other avian influenza viruses resulting in a diversity of H5Nx viruses^1^ with different types of neuraminidase (mostly N5, N6, N8, and N2), while conserving the H5 genes (4). The nomenclature of H5Nx viruses that clustered in this divergent HA group was updated as a new clade, 2.3.4.4, in addition to two other new clades (5). Up and until 2014, these reassortants were only found in East Asia (China, South Korea), but during the winter 2014–2015, the H5N8 virus started to spread into the United States and Europe. This range expansion was unprecedented and contrasted with the path that had been previously followed by HPAI H5N1. In 2006, the first long-distance intercontinental spread of HPAI H5N1 seemed to involve several stepping-stones of key migration stopover sites such as Qinghai Lake in central China (6), the Omsk and Novosibirsk region in Siberia, and the Black Sea basin in Romania and Turkey (7). At the time, introduction of HPAI H5N1 in the Black Sea basin was followed by a wave of expansion into western Europe linked to a cold front pushing large populations of potentially infected waterfowl along the 0°C frost isocline (8). Under similar conditions, a second spread of HPAI H5N1 clade 2.3.2.1 into eastern Europe was observed in 2010 (9).

In contrast, the range expansion of HPAI H5N8 clade 2.3.4.4 into Europe and the United States was reconstructed through phylogeographic inference and was apparently very different (10). It did not pass through these central Asia migration stopover sites, but apparently, it involved wild waterfowl moving from breeding regions in the Arctic spreading south into Europe and North America. In addition, the long-distance spread of HPAI H5Nx clade 2.3.4.4, which was first documented in the winter 2014–2015, apparently repeated itself in the winter 2016–2017. The second transmission mainly affected Europe and caused numerous HPAI H5N8 poultry outbreaks.

Thus, novel introductions of HPAI H5Nx clade 2.3.4.4 into Europe and the United States originated from changes in the epidemiology of HPAI viruses taking place in Asia. By comparing the spatial and temporal distribution of the HPAI H5Nx records in Asia, we observed three noteworthy observations (Figure 1). First, in the period from 2004 to 2011, the HPAI H5N1 virus represented a large majority of all HPAI outbreaks and large-scale epizootics across much of Asia. Second, in the period from 2012 to 2017, the emergence of new H5N8, H5N6, and H5N2 subtypes mainly involved China and South Korea where these new subtypes represented the majority of outbreaks. In contrast, although some of these new subtypes were introduced into southeast Asia (e.g., H5N6 in Vietnam), so far, the large majority of outbreaks have been caused by HPAI H5N1 viruses. Third, while there is a decreasing trend of HPAI H5N1 outbreaks in south and southeast Asia, emergence of these new subtypes in China and South Korea have caused very significant epizootics leading to an increase in the total number of HPAI outbreaks in comparison to the 2004–2011 period. Those differences may indicate why long-range transmission of HPAI H5N1 viruses have changed, because changes in disease circulation in different regions have created different patterns of transmission across the rest of the world. From 2004 to 2011, the majority of outbreaks were in southeast and south Asia, and long-range transmission through migratory birds from south Asia would require transmission through the Central Asian Flyway. From 2012 to 2017, the proportions changed, with comparatively more outbreaks located in east Asia caused by emergence of new clade 2.3.4.4 reassortants, and higher chances of long-range transmission through the Australasian-East Asian Flyway. In both cases, transmission into Europe or the U.S. by migratory birds likely would require movement through breeding areas in the Arctic. The migration routes used and the set of species involved may have changed, and with those changes, the spatiotemporal pattern of introduction risk into those distant regions was altered.

**Figure 1:**
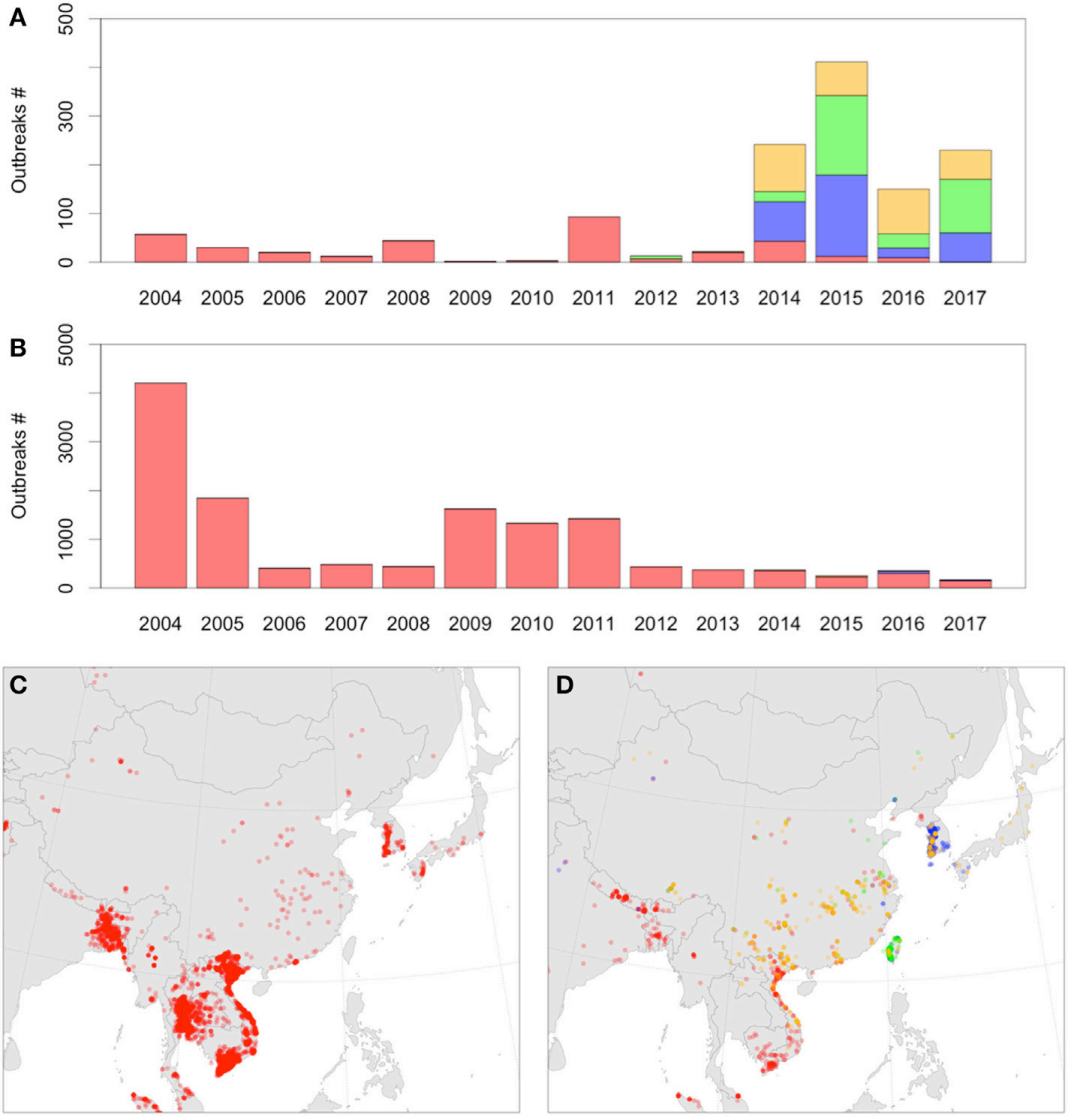
**(A)** Number of highly pathogenic avian influenza (HPAI) H5Nx domestic outbreaks in China, North Korea, South Korea, and Japan over time **(A)** compared to all other Asian countries **(B)**, colored by subtype: H5N1 (red), H5N2 (green), H5N8 (blue), and H5N6 (orange). **(B,C)** Distribution of HPAI H5Nx domestic outbreaks in Asia in 2004–2011 **(C)** and 2012–2017 **(D)** with the same color code (extraction from the Empres-I database from first Jan 2004 to 13th June 2017, all H5Nx HPAI outbreaks).

Many datasets have been collected over the past decade documenting the migration of wild waterfowl in Asia (Figure 2). Several previous studies have investigated specific possible long-range transmission events of HPAI H5N1 viruses with some of these datasets (11–15) and highlight how migration patterns sometimes matched the distribution of cases in space and time, but also providing examples where they did not. When pooled together, these data illustrate the broad-scale pattern of the two important flyways passing through Asia. The Central Asian Flyway connects western Mongolia to South Asia and Myanmar, while the Australasian-East Asian Flyway connects eastern Russia, South Korea, Japan, and China. A recent phylogeography study also showed that inferred viral migration structure correlated well with large-scale wild bird migration (16), but the authors did not test alternative transmission structures such as trade. If we focus on the Australasian-East Asian Flyway, we can see many potential connections between China and South Korea. Connectivity from the south involves long-distance migrants with linkages from Guangdong (birds marked in Hong Kong) or Jiangxi Provinces (birds marked at Poyang Lake). These connections and their possible role in transmitting HPAI H5N1 viruses over long distances have been described in detail in previous work (12, 17).

**Figure 2:**
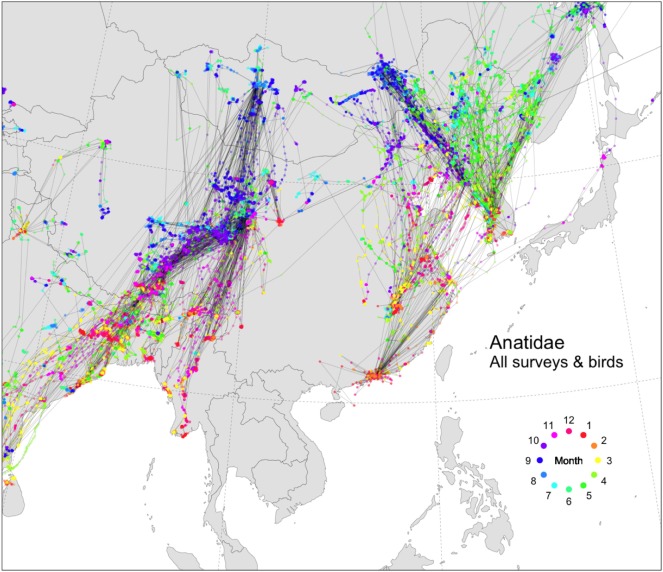
Tracks of wild Anatidae in and around China, colored by month (524 birds, 19 species, from 2006 to 2017, USGS/FAO/KoEco/APQA/NIER surveys). *Anas penelope* (*n* = 33), *Anas acuta* (*n* = 50), *Anser indicus* (*n* = 98), *Anas strepera* (*n* = 17), *Tadorna ferruginea* (*n* = 51), *Anas crecca* (*n* = 20), *Anas querquedula* (*n* = 16), *Anas clypeata* (*n* = 19), *Anser anser* (*n* = 2), *Anas platyrhynchos* (*n* = 98), *Anas poecilorhyncha* (*n* = 29), *Anas formosa* (*n* = 2), *Anas falcata* (*n* = 5), *Netta rufina* (*n* = 1), *Anser cygnoides* (*n* = 46), *Cygnus cygnus* (*n* = 11), *Cygnus columbianus* (*n* = 2), *Anser albifrons* (*n* = 22), *Anser fabalis* (*n* = 2). The data presented here were collected according to handling and marking procedures approved by the USGS Patuxent Wildlife Research Center Animal Care and Use Committee and the Animal and Plant Quarantine Agency ethical committee. The different datasets are described in the Movebank database (https://www.movebank.org).

These data also may be examined in relation to the risk of HPAI H5Nx viruses in poultry. A recently published spatial model of HPAI H5Nx suitability (Figure 3) shows an overlay with tracks of wild migratory birds. Along the Central Asia Flyway, wild bird tracks do not necessarily connect high-risk areas together (Figure 3). Rather, high-risk areas from south Asia are connected to regions in China where wild birds may be abundant, and potentially infected, such as at Qinghai Lake, but where the risk of HPAI H5Nx transmission in poultry is predicted to be low, mostly because of very low densities of poultry farming. In contrast, the East Asian Flyway links many hotspots of high suitability for HPAI H5Nx infection: the Hong-Kong and Guangdong area, the Poyang Lake area, the Shanghai area, the western coasts of North and South Korea, and minor hotspots of high suitability located in northeastern China. China, North and South Korea, and to a lesser extent Japan, seem to be part of an interconnected epidemiological landscape that has been particularly active in the last few years, with the emergence of several reassortants causing serious HPAI epizootics. Circulation of HPAI H5Nx viruses in any one of these interconnected areas may potentially impair efforts made in others to control or eradicate the disease, and calls for more close collaboration to address the problem regionally (18, 19).

**Figure 3:**
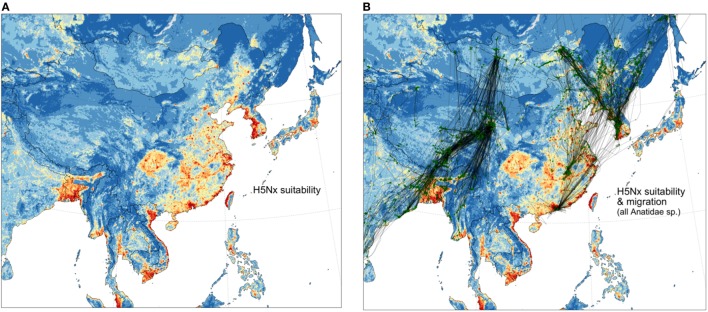
Distribution of suitability for infection by HPAI H5Nx viruses from Dhingra et al. (20) spatial model **(A)** and spatial overlay with all Anatidae migration tracks **(B)**.

A particular focus can be put on the many apparent connections between South Korea and the northeastern China provinces of Jilin, Heilongjiang, and Liaoning, because these three provinces have been subject to relatively rapid land-use changes in the last decade. Figure 4 shows the spatial distribution of rice paddy in 2015 and the changes that took place in % of rice paddy cover between 2000 and 2015 as quantified by remote sensing with MODIS images at 500-m spatial resolution (21). Vast areas were transformed into rice paddy fields in northeastern China, in particular, in the province of Heilongjiang (from 1.28 to 4.48 million ha), and to a lower extent, Jilin (from 0.58 to 0.68 million ha), while there was a minor reduction in Liaoning (from 0.75 to 0.66 million ha), as shown in Figure 5. Those changes are ecologically relevant, summing up to 3.21 million ha (21). In comparison, the harvested rice paddy area in nearby countries was 908,194 ha in Korea in 2015 according to the Korean Statistical Information Service, and 1,575,000 ha in Japan in 2014 according to FAOSTAT (22), and the rice areas in those countries showed a decreasing trend during the same period (22).

**Figure 4:**
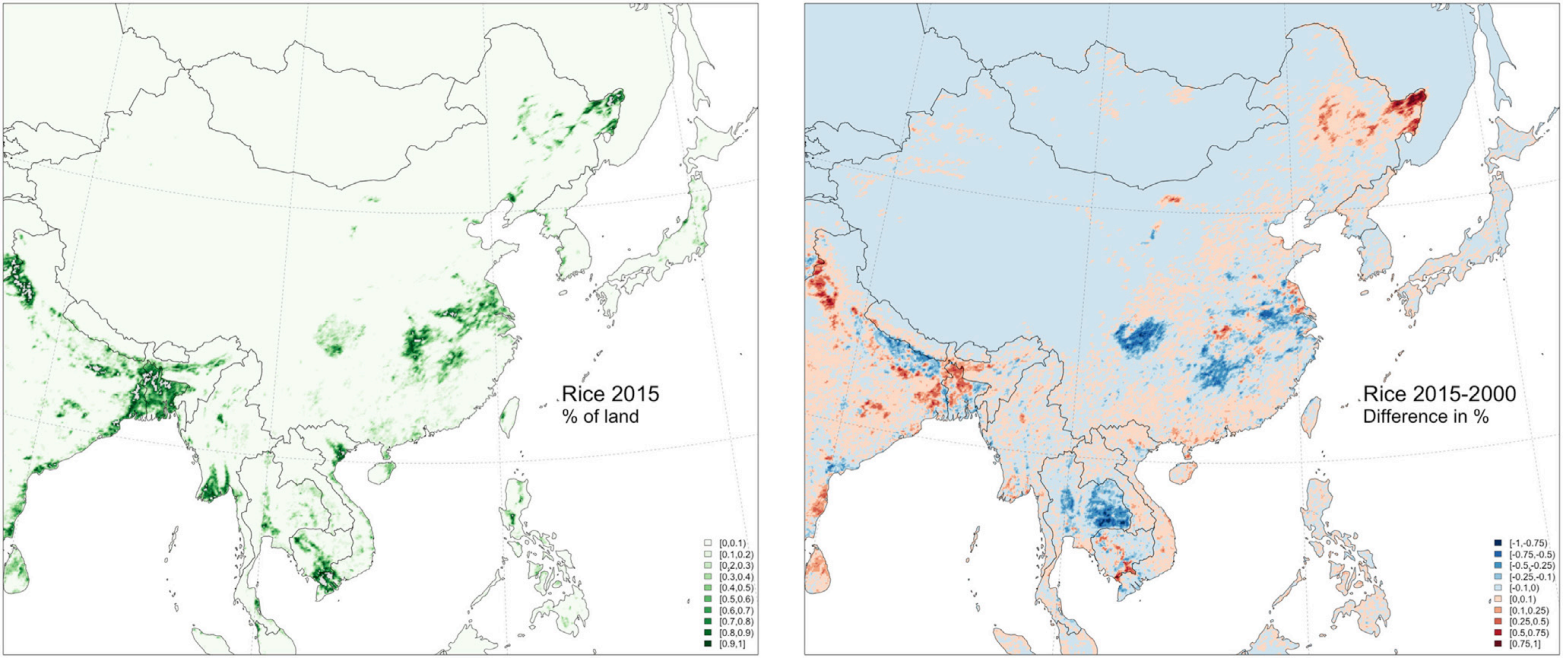
Distribution of rice harvest in 2015 (left) and % difference between the surface harvested in 2015 and 2000 at 5 km × 5 km grid cells, according to Zhang et al. (21).

**Figure 5:**
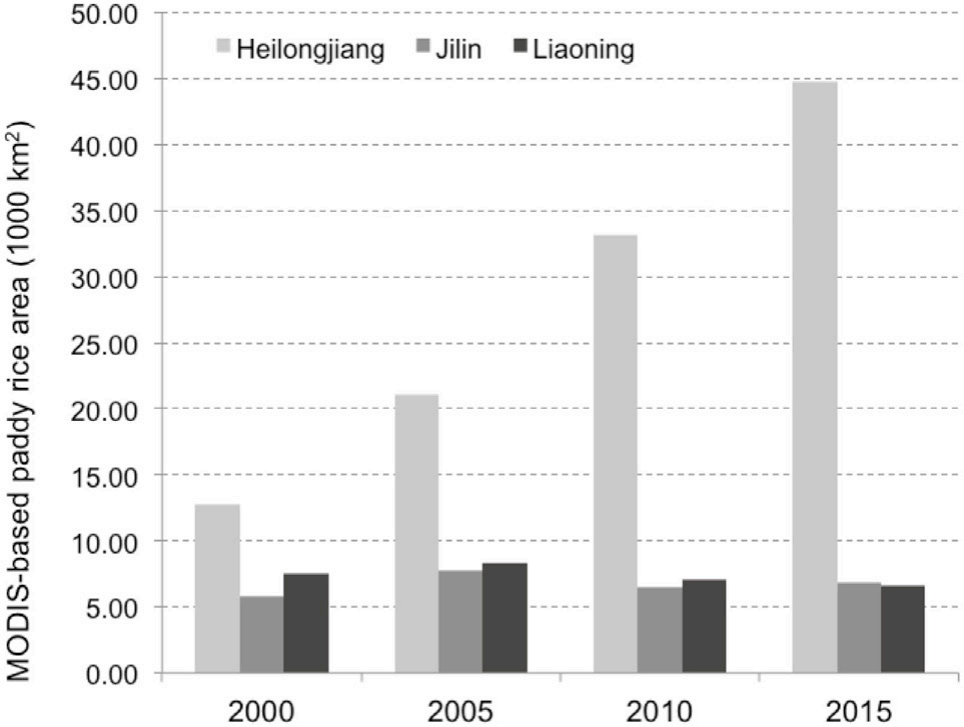
Temporal distribution of rice harvested areas in different Chinese provinces over time, according to Zhang et al. (21).

Rice paddy fields occupy a major role in the ecology of avian influenza, because inundated paddy fields serve as artificial wetlands that are ideal habitats for many waterfowl (23, 24). Furthermore, in many instances, rice paddy fields have been planted in or next to former natural wetlands used by waterfowl to take advantage of the water supply. Paddy fields represent attractive habitats for many species of waterbirds that can be considered as pests if they feed on plants, or they may simply forage on leftover grain in post-harvested paddy fields before the next planting season with no impact.

In many parts of Asia, post-harvested paddy fields are also used to feed free-grazing domestic ducks (25, 26). These free-grazing ducks were previously found to be a very important risk factor for the transmission of HPAI H5N1 viruses (27, 28). Free-grazing duck use of the same habitat as wild waterfowl can present many opportunities for indirect transmission as was observed in Poyang Lake (17, 26) and Dongting Lake and other wetlands along the lower Yangtze River wetlands with similar agro-ecosystems associating rice and duck farming. Conversely, free-grazing ducks also may forage in natural wetlands in the Poyang Lake area (26). This association between rice paddy fields and free-grazing ducks is common throughout Asia. However, most observations have been in more southern latitudes including Poyang Lake (17), the Thailand central plain (25), the Vietnam deltas (29, 30), Bangladesh, or Indonesia (31); however, the extent of this association in the newly planted areas of north-eastern China is unknown.

Prosser et al. (32) developed a model to map transmission risk between wild waterfowl and domestic poultry in China including during the wintering and breeding seasons (Figure 6). The transmission risk during the wintering season includes the likely source area for the HPAI H5N1 virus, which was previously identified in several spatial risk modeling studies (33, 34) as the Yangtze and Pearl River systems. In addition, transmission risk at this interface identifies broad areas in northeastern China in the same provinces of Jilin and Heilongjiang that were identified to be highly connected to South Korea (Figure 2) and that underwent rapid land use changes toward rice cropping (Figure 4). The area also contains several minor hotspots suitable for HPAI H5Nx transmission in domestic poultry (Figure 3) where a few HPAI H5N6, H5N8, and H5N2 poultry outbreaks were documented in the period from 2012 to 2017 (Figure 1).

**Figure 6:**
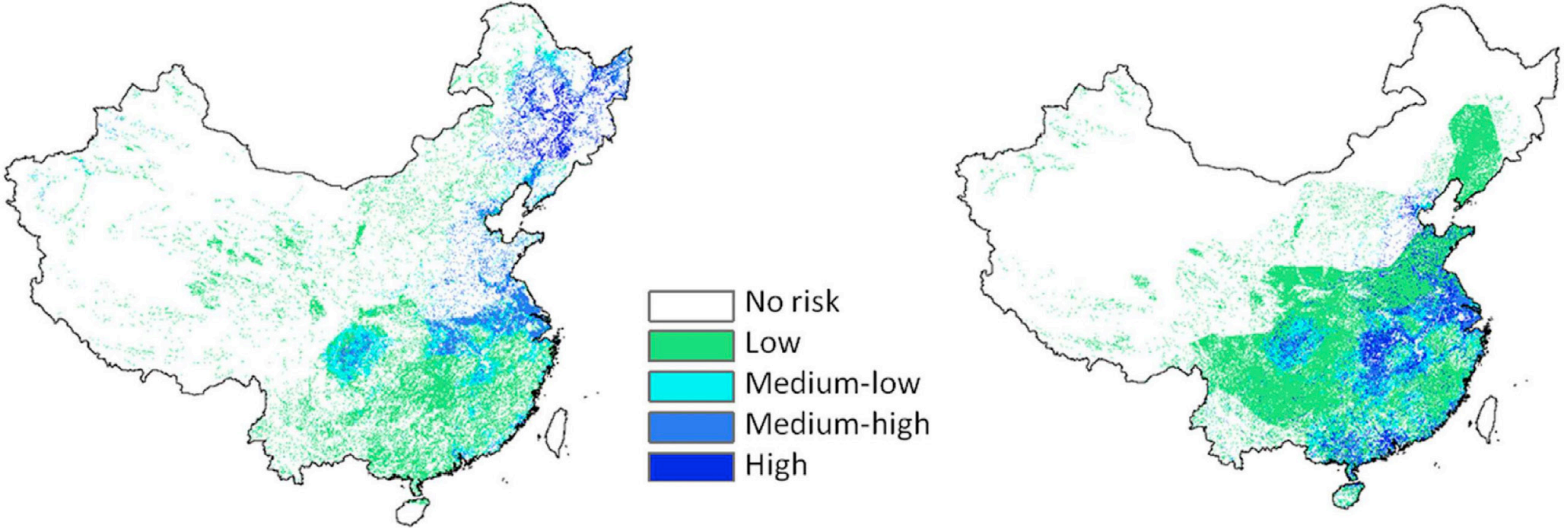
Risk of domestic-to-wild avian influenza transmission during the breeding (left) and wintering (right) season according to Prosser et al. (32).

Thus, we hypothesize that recent land-use changes in northeastern China (Figure 4) may have influenced the spatial patterns of transmission of avian influenza at the regional and inter-continental scale. In terms of the suitability of chronology for transmission, the main harvest of the rice in this part of northeastern China is from September to October. During this period, long-distance migrants use these areas as stopover sites after leaving the breeding grounds and short-distance migrants may still be present before migrating to southern latitudes as the winter approaches. Stopover sites involve concentrations of many species with large numbers of immunologically naïve juveniles alongside adult birds, forming an ideal setting for AIV transmission and redistribution. Previous work on low pathogenic AIV (LPAIV) ecology have shown that autumn concentrations of waterfowl with high recruitment rates of immunologically naïve juveniles induce a seasonally and geographically distinct pattern in LPAIV prevalence with a marked peak in the autumn (35–37). The mechanisms of the seasonality in LPAIV prevalence involve annual replenishment of susceptible juveniles together with the introduction of new viruses by migrants (38), and this translates into large-scale spatial gradients of LPAIV that are apparent at a continental scale (39). By the same mechanisms, areas recently converted into rice paddy fields may have become important stopover sites with high transmission of HPAI viruses between different species and populations prior to their southern migration. The suitability of those areas as stopover sites with high transmission potential may further be amplified by the coincident timing of the rice harvest, which leaves a significant amount of leftover grain on the ground (17, 23, 24). Development of new stopover sites with high transmission potential resulting from land-use change could have broad scale implications. Waterfowl stopping in these areas (Figure 2) may continue their migration into North and South Korea as well as eastern and southern China, increasing the risk of long-distance transmission of any AIV acquired in these stopover sites.

The extent of the domestic-to-wild and wild-to-domestic interface in the area is poorly known and to our knowledge, wild water bird count data are not available for those newly planted areas. At broad scale, the highlighted models (Figures 3 and 6) and reports of HPAI H5Nx poultry outbreaks in the area (Figure 1) suggest that there could be a risk of transmission between these populations. Investigations at finer spatial and temporal scales would be needed to explore more thoroughly the role that these new rice planted areas may have played in this regard. Similarly, better documenting the role played by the changes in land use on intercontinental spread would require investigating habitat use and foraging activities during the spring migration at the time of rice planting rather than harvest. Finally, avian influenza surveillance in China is based on a combination of passive surveillance in poultry farms reporting HPAI outbreaks, sero-surveillance survey designed to routinely assess vaccination coverage, and active surveillance made in live-poultry markets (33). However, there are relatively few live-poultry markets reported in those areas compared to more southern parts of China (40). So, the combination of mass-vaccination in poultry farms, and of relatively low live-poultry market surveillance may combine to lead to a low detection capacity, and further work would be needed to better document the possible circulation of avian influenza viruses in those northeastern areas.

Several approaches could be envisaged to investigate the hypothesis outlined above. First, detailed studies of the use of different types of habitats by the waterfowl during the spring migration, breeding period, and autumn migration would document how these rice areas are actually used in different seasons. Second, a better characterization of the domestic–wild interface through the collection of data on poultry distribution and movements in these areas would be needed to quantify possible links with the circulation of HPAI H5Nx viruses in poultry. Third, sampling of AIV viruses in and around these stopover sites and phylogeographic analysis may help to characterize their prevalence in the epidemiological system of northeastern Asia.

## Ethics Statement

The data included in this paper regarding wild bird migration tracking were collected according to handling and marking procedures approved by the USGS Patuxent Wildlife Research Center Animal Care and Use Committee (USA) and the Animal and Plant Quarantine Agency (South Korea) ethical committee.

## Author Contributions

MG drafted the first manuscript. DP, SN, JT, GZ, XX, WJ, and HL provided data and all authors contributed to the discussions and editions of the final manuscript.

## Conflict of Interest Statement

The authors declare that the research was conducted in the absence of any commercial or financial relationships that could be construed as a potential conflict of interest.

## Footnotes

^1^This paper deals only with H5Nx viruses and specifically does not address the evolution and spread of H7N9 virus within China.
